# Installation and imaging of thousands of minirhizotrons to phenotype root systems of field-grown plants

**DOI:** 10.1186/s13007-022-00874-2

**Published:** 2022-03-27

**Authors:** Ashish B. Rajurkar, Scott M. McCoy, Jeremy Ruhter, Jessica Mulcrone, Luke Freyfogle, Andrew D. B. Leakey

**Affiliations:** 1 Institute for Genomic Biology, University of Illinois at Urbana-Champaign, Urbana, IL USA; 2 Department of Plant Biology, University of Illinois at Urbana-Champaign, Urbana, IL USA; 3 Department of Crop Sciences, University of Illinois at Urbana-Champaign, Urbana, IL USA

**Keywords:** Minirhizotron, Root, Field, Phenotyping, High Throughput phenotyping

## Abstract

**Background:**

Roots are vital to plant performance because they acquire resources from the soil and provide anchorage. However, it remains difficult to assess root system size and distribution because roots are inaccessible in the soil. Existing methods to phenotype entire root systems range from slow, often destructive, methods applied to relatively small numbers of plants in the field to rapid methods that can be applied to large numbers of plants in controlled environment conditions. Much has been learned recently by extensive sampling of the root crown portion of field-grown plants. But, information on large-scale genetic and environmental variation in the size and distribution of root systems in the field remains a key knowledge gap. Minirhizotrons are the only established, non-destructive technology that can address this need in a standard field trial. Prior experiments have used only modest numbers of minirhizotrons, which has limited testing to small numbers of genotypes or environmental conditions. This study addressed the need for methods to install and collect images from thousands of minirhizotrons and thereby help break the phenotyping bottleneck in the field.

**Results:**

Over three growing seasons, methods were developed and refined to install and collect images from up to 3038 minirhizotrons per experiment. Modifications were made to four tractors and hydraulic soil corers mounted to them. High quality installation was achieved at an average rate of up to 84.4 minirhizotron tubes per tractor per day. A set of four commercially available minirhizotron camera systems were each transported by wheelbarrow to allow collection of images of mature maize root systems at an average rate of up to 65.3 tubes per day per camera. This resulted in over 300,000 images being collected in as little as 11 days for a single experiment.

**Conclusion:**

The scale of minirhizotron installation was increased by two orders of magnitude by simultaneously using four tractor-mounted, hydraulic soil corers with modifications to ensure high quality, rapid operation. Image collection can be achieved at the corresponding scale using commercially available minirhizotron camera systems. Along with recent advances in image analysis, these advances will allow use of minirhizotrons at unprecedented scale to address key knowledge gaps regarding genetic and environmental effects on root system size and distribution in the field.

## Background

Roots play key roles in anchoring plants to their substrate, acquiring resources from the soil, and as a link between the plant and abiotic or biotic components of the belowground environment [38, 35, 13, 30]. As a result, root system size and distribution significantly impacts plant productivity, sustainability and resilience to stress [28] and root traits have been identified as key targets for crop improvement [29].

Despite the importance of root systems, many key questions related to genetic and environmental variation in their development, productivity and function remain unanswered because being buried in soil makes them very difficult to measure [2, 46, 55, 56]. There have been important recent advances in root phenotyping that range from rapid phenotyping of many plants under controlled environment conditions to more arduous, often destructive, methods applied to relatively few plants grown under field conditions [2, 46, 48, 55].

Transparent growth media in containers allows time courses of 2D or 3D root imaging with digital cameras [19, 31, 45]. Greater realism has been achieved while remaining in controlled environment conditions through the use of opaque artificial media [14, 27, 34], or soil in pots and other imaging modalities, including X-ray computed tomography (X-ray CT) [32], magnetic resonance imaging (MRI) [41, 49], positron emission tomography (PET) [16], and neutron radiography [55].

In the field, the trade-off between the realism of the growing environment and the rate or scale of data collection has proven hard to break, with studies of significant genetic diversity in the field most often being limited to destructive measurement of the root crown towards the end of the growing season [12, 47]. Deep roots play a pivotal role in plant function, including acquisition of nutrients and water [26].

Assessing the size and depth distribution of the entire root system of field-grown crops is still only possible with three technologies that have been in use for decades. First, soil cores can be collected and roots washed out in different layers through the soil profile, but this is destructive and very laborious (e.g. [5, 24]. The core-break method [15] can be used to screen tens of genotypes [53] and recently has been made more efficient through innovations in image acquisition and analysis [54], but it also requires destructive sampling at the end of the field season. Third, minirhizotrons can be used to non-destructively image the size and depth distribution of root systems at different times in the growing season at tens of locations within an experimental field (e.g. [3, 8, 17, 18]). Minirhizotrons installed at an angle of 30–45˚ from vertical are most commonly recommended for use in an open field setting because vertical tubes can cause artifacts in root distribution [7, 21], [37]. Horizontal tubes have valuable applications, but require trenching to allow installation, and tend to be used in specialized facilities (e.g. Wahlstrom et al. 2021). Therefore, angled minirhizotrons were the focus for developing new methods of rapid installation suitable to general use in crop field trials.

This study aims to facilitate a breakthrough in high-throughput phenotyping of root systems in the field by developing and refining methods that allow thousands of minirhizotrons to be deployed in a single field experiment. This is a significant challenge because minirhizotron installation cannot start until after a crop is sown, otherwise the portion of the minirhizotron tube protruding above the soil surface would interfere with the operation of a mechanical planter. In addition, installation of access tubes must be complete before the crop has germinated and grown to a stage where installation would damage plants. In a field setting, this period lasts only a few weeks. The unpredictability of weather patterns in many locations means that field activities are unlikely to be possible every day even if installation is possible under a range of soil moisture contents.

Minirhizotron tubes can be installed after excavating holes with hand augers [1], hand held power augers [23], and augers or hydraulic soil corers mounted to vehicles, including tractors [17]. All of these methods of tube installation have been applied for several decades [6, 8]. Method development has focused heavily on the best material to use for minirhizotron tubes and ensuring good quality installation i.e. minimal soil disturbance and close contact between the soil and outer surface of the tube throughout its length [21]. While this is essential, the scale of experiments has remained limited, with only tens of minirhizotrons being installed in each experiment, even when vehicle/mounted corers were used for installation in relatively easy to access agricultural systems (e.g. [6, 10, 17, 18, 33, 57]). This study built on 10 years of experience using traditional approaches to install minirhizotron tubes. Methods for using tractor-mounted hydraulic corers were modified and accelerated. The use of multiple systems in parallel to scale-up operations was also demonstrated. Finally, we tested if currently available minirhizotron cameras can be used to collect images in a reasonable timeframe when the scale of an experiment increases to use thousands of minirhizotrons.

## Results and discussion

**Fig. 1:**
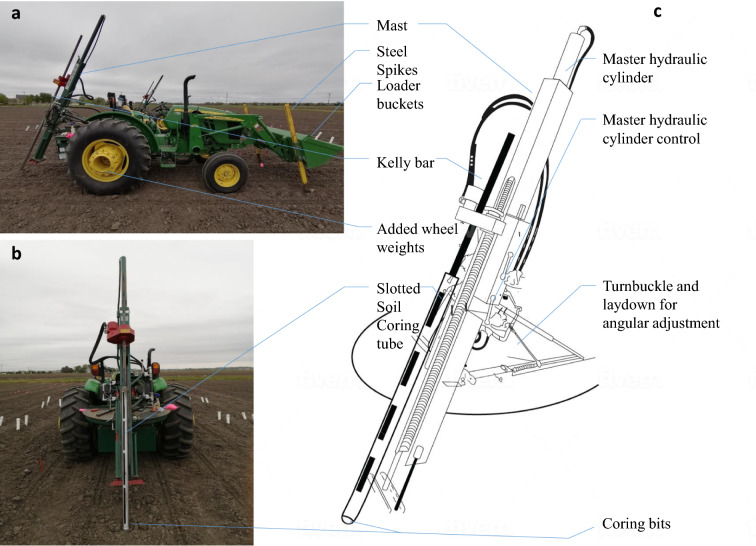
Tractor-mounted Giddings probes shown as: **a** side view photograph; **b** rear view photograph; and **c** diagram of key components and modifications

Methods were successfully developed to install and collect images from 1508—3038 minirhizotron tubes in a single experiment in each of three successive growing seasons (Tables 1 and 2; https://www.youtube.com/watch?v=kciMoWH-fFk). The commercially available tractor-mounted hydraulic corer that was used is designed for sampling vertical soil cores (Fig. 1). It was modified with a telescoping steel laydown bar to fix the angle of the corer at 30˚ from vertical (Fig. 1). This was used to bore a ~ 152 cm-long hole in the ground of the same diameter as the cellulose acetate butyrate (CAB) minirhizotron tube. To avoid soil compaction and accelerate installation, multiple short cores were extracted in succession from each hole. The coring tube was removed after each short core was collected and replaced with a new coring tube. This allowed the next core to be excavated while the soil from the previous coring tube was collected into a bucket. This approach was especially important when soils were wet because it greatly reduces how often the soil core becomes stuck inside the coring tube. Wet soil blocking a series of coring tubes can completely halt minirhizotron installation, and must be avoided. For the same reason, slotted coring tubes that were easier to clean out and coring bits with increasingly small internal diameters (i.e. “quick relief” bits) were used when soils were wetter. After boring the hole, the minirhizotron tube was inserted by placing the PVC cap over the tube and pushing down on it with the base of the soil tube adapter of the hydraulic system. All excavated soil was transported to the boundary of the field in order to avoid altering soil properties near the minirhizotron tubes. The PVC plastic cap covered the portion of the tube protruding from the soil completely, thereby serving to block light transmission that could otherwise alter root growth [21, 25] and blocking rain from entering the tube.

**Table 1:** Year, tubes installed, days required and efficiency of installation method

Year	Planting Date	Beginning of Tube Installation	End of Tube Installation	Plots with Tubes	Tubes Installed	Average Tubes per Plot	Installation Days	Average Tractors Used Per Day	Operators per tractor	Total Worker days	Average Tubes Installed per Worker Hour	Average Tubes Installed per 8 h per Tractor
2017	5/31/2017	6/5/2017	6/13/2017	754	1508	2.00	7	4	2	56.0	3.37	53.9
2018	4/29/2018	4/30/2018	5/29/2018	950	3015	3.17	13	3.333	2	86.7	4.35	69.6
2019	5/18/2019	5/20/2019	6/7/2019	783	3038	3.88	9	4	2	72.0	5.27	84.4

**Table 2:** Year, tubes imaged, images collected, days required and efficiency of imaging

Year	Beginning of Tube Imaging	End of Tube Imaging	Tubes Imaged	Images Collected	Average Tubes per Plot	Imaging Days	Average Cameras Used Per Day	Operators per Camera	Total worker days	Average Tubes Imaged per Worker Hour	Average Tubes Imaged per 8 h per camera
2017	8/7/2017	8/17/2017	1053	109,473	2.00	9	2.33	1	21.0	6.28	50.2
2018	7/17/2018	8/6/2018	2966	319,726	3.17	16	3	1	48.0	7.72	61.8
2019	7/30/2019	8/14/2019	2872	295,971	3.88	11	4	1	44.0	8.16	65.3

The forces required to bore out the hole and insert the minirhizotron tube are considerable, and can be sufficient to lift the tractor off the ground and also push it forwards. If this happens, it can cause the minirhizotron tube to bend and not have good contact with the soil down its entire length. It can also cause damage to equipment and can slow down installation. Therefore, 350 kg of weights were added to the rear wheels of the tractor (Fig. 1). In addition, custom-made steel spikes were attached to the front loader so that they could be inserted up to 50 cm into the soil as a physical brake against forward motion during installation.

**Fig. 2:**
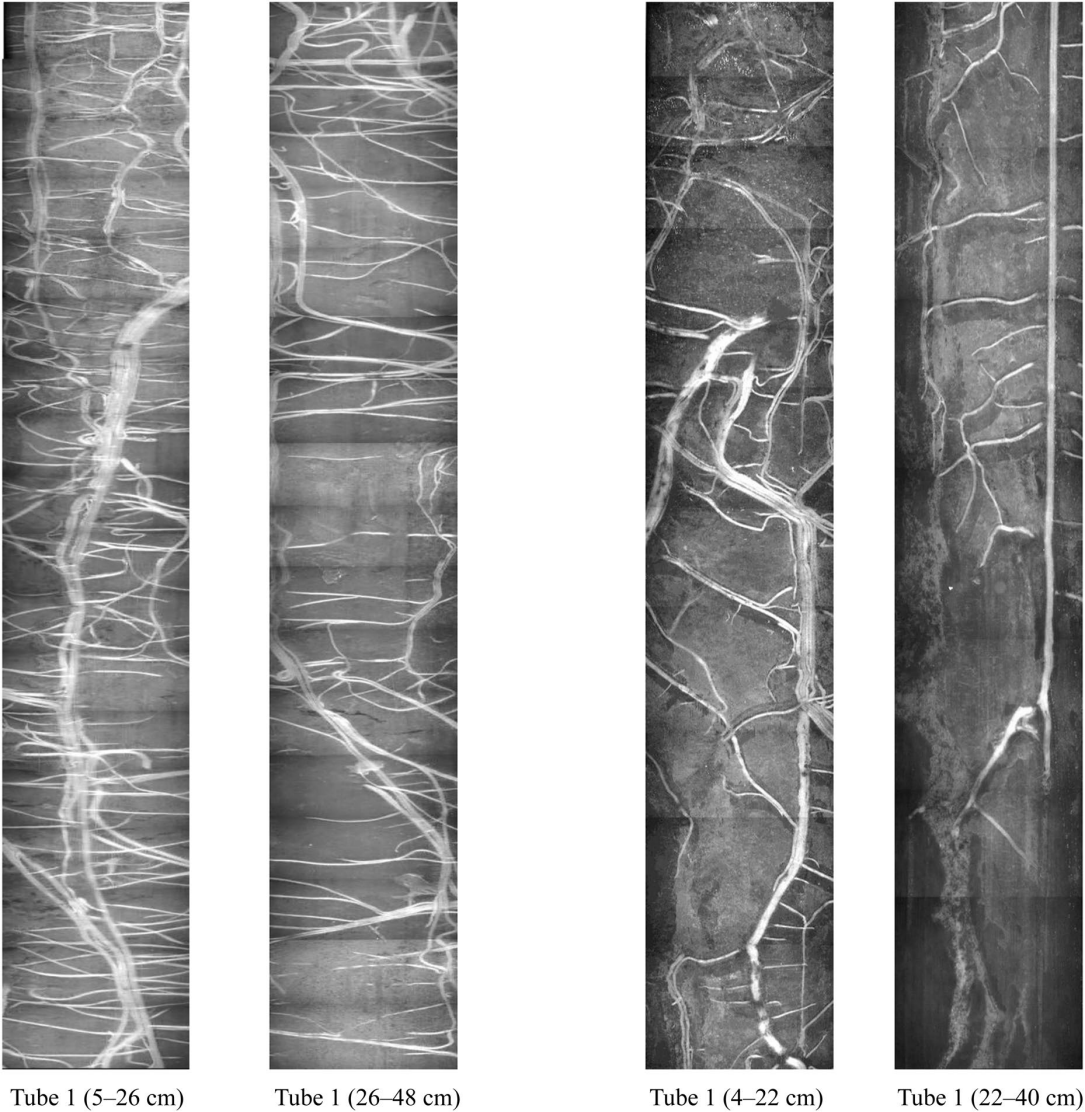
Examples of images from portions of the soil profile from two example minirhizotron tubes showing variation in the root system size and distribution at shallow to moderate depths. Individual images with dimensions of 18 × 13 mm were stitched together using Adobe Photoshop

The quality of root observations made in minirhizotrons is strongly influenced by the method of installation [9]. The main goal is to make good contact between the surrounding soil and the entire length of the tubes. This requires straight holes to be bored with precision [21]. This was aided by the use of a tractor-mounted hydraulic corer with steel coring tubes and tractor weights/stabilization. If this was not achieved, excessive root proliferation would be observed in gaps between the soil and tube surface, as has been reported in some prior occasions [22, 40, 51]. However, this was not the case and roots were distributed along the tube in accordance with expectations (Fig. 2, [4, 20]. In addition, radial soil cracks can form in surface soil around the tube causing unwanted changes in root distribution [40], but this was not observed in the current study.

**Fig. 3:**
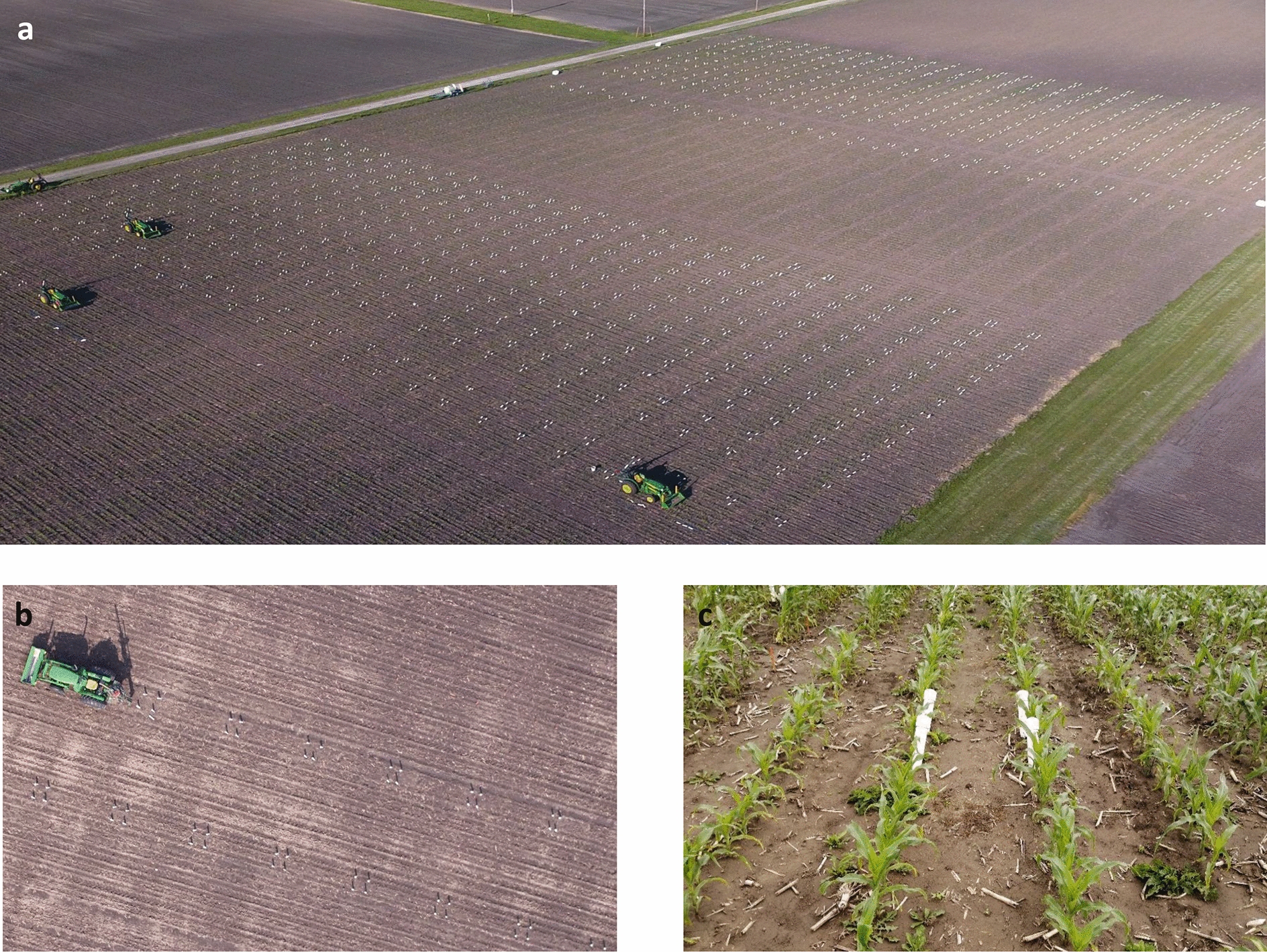
Large scale minirhizotron installation in 2019, demonstrating capacity of installation with the presented method, as **a** aerial photograph of full experiment, **b** aerial photograph of portion of the experiment showing four minirhizotron tubes per subplot, **c** close up from ground of a single subplot showing PVC caps on tubes and normal plant growth at a later growth stage

Progressive refinement of methods to produce the technique described above allowed the average rate of tube installation per worker per hour to increase from 3.37 in 2017 to 4.35 in 2018 and 5.27 in 2019 (Table 1). This translated into each tractor installing 53.9 to 84.4 tubes on average per day in a given year (Table 1), although a single tractor regularly succeeded in installing > 100 tubes in a day. On a typical day, four tractors with hydraulic corers were operated in parallel by a team of eight people i.e. two people per tractor. In the final year, this allowed 3038 minirhizotron tubes to be installed in a single experiment (Table 1; Fig. 3) over 9 days of work that were completed within 17 days after sowing. The length and angle of tube protruding from the soil was quickly and easily measured after installation. The tubes were deployed in 783 plots, with 3.88 tubes per plot on average, allowing root system size and distribution to be assessed across hundreds of genotypes of maize.

Operating at such a large scale (i.e. > 5.5 km of total minirhizotron tubing in a single experiment) requires logistical planning to support the storage, transport and distribution of materials. Minirhizotron tubes, minirhizotron caps and drilling supplies were transported to the edge of the field by forklift and then the materials needed for a day’s work were stored in the front-loader bucket of the tractor, saving considerable manual labor.

The soils at the study site used here are silty clay loams of the Drummer-Flanagan soil series. Consequently, they are deep (> 1 m), contain almost no stones or rocks, and the walls of the holes created by the soil coring equipment are stable. As such, they are an ideal location for any method of root phenotyping, including minirhizotrons. And, they are found on millions of acres of farmland in the Midwestern U.S., making them representative of the region. The methods described here have not been tested in other locations as yet. But, wherever similar soil conditions exist they are likely to be suitable. Minirhizotrons have been used in smaller numbers to investigate root systems in a wide range of soil types (e.g. [36]) through modification of installation protocols. In very sandy soils, holes tend to collapse after boring. In stony soils, holes tend to be very difficult to bore out and have very uneven shapes, leading to poor contact between the minirhizotron tubes and the soil. In both cases, holes larger than the diameter of the tube can be bored and then back-filled once the tube has been installed [42]. These steps slow installation sufficiently that very large installations of thousands of minirhizotrons would likely be unrealistic under such conditions. But, even then, the methods described here could be applied to more modest experimental designs and would still result in significantly less physical effort and more efficient installation than traditional manual methods.

**Fig. 4:**
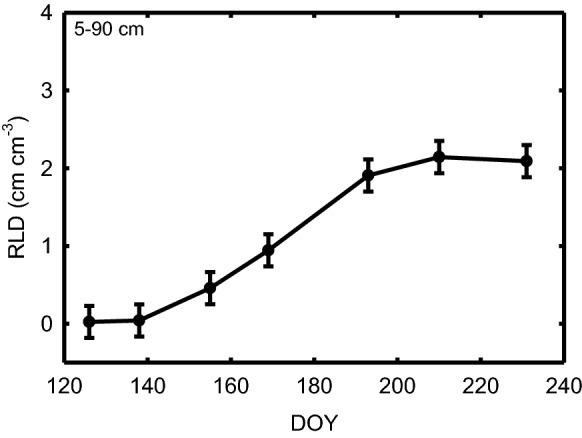
Total root length density (RLD; cm cm-3) of maize at depths of 5–90 cm on mutliple days of year (DOY). Data was collected using the methods of Gray et al. [18], including manual analysis of all images. Data shown are lsmeans ± standard errors.

Increasing the scale of minirhizotron installation to such a large degree potentially means other aspects of using this root phenotyping approach become unfeasible. Fortunately, tools for automated analysis of images from minirhizotrons are becoming available [43, 57, 58]. But, image acquisition must also be feasible in a reasonable timeframe. We demonstrated that with four commercially available minirhizotron cameras being used in parallel, the large number of minirhizotrons that were installed could be imaged within 9–16 days when starting immediately after anthesis. This represents a period in time when prior experiments indicated that maize root system size and depth has reached its maximum and is stable prior to the onset of senescence at the end of the growing season (Fig. 4). The average number of tubes imaged per day by each camera operator increased from 50.2 in 2017, to 61.8 in 2018, and 65.3 in 2019 (Table 2). In 2017, only 70% of the installed tubes were imaged because drought and heat during and immediately after planting resulted into poor establishment of experimental plots and the plots with poor establishment were excluded from imaging.

This approach means that phenotyping genetic and/or environmental variation in final root system size and distribution is immediately possible. While that does address an important knowledge gap, it would still not be feasible to collect data across all minirhizotrons at other periods of the growing season when the root system is growing at a rapid rate. That limits the ability to test for variation across time in the effect of environmental treatments or genetic variation [17, 18]. And, it prevents rates of root system growth (e.g. Figure 4) from being measured, which is significant because data on genetic variation in early-season rates of above-ground growth have proven very valuable in predicting end-of-season biomass production [50]. Accelerating the rate of image acquisition could be achieved by: (1) a robot or robots that move around the field visiting minirhizotrons and collecting data, (2) cameras that can acquire images automatically when installed permanently in each minirhizotron, or after being moved from minirhizotron to minirhizotron [11, 39, 44], (3) or by a large team of people using many cameras in parallel. All of these options will require further innovation and/or commercialization to make the necessary equipment available and affordable.

It is important to highlight that all root phenotyping methods have strengths and weaknesses. Consequently, whenever two or more approaches can be applied simultaneously, the quality of the data is likely to improve and the number root system traits will increase. For example, the shovelomic method of phenotyping root crowns provides rapid and detailed evaluation of a large proportion of crop roots in shallow soil layers [12, 47]. This is highly complementary to the information on root system size and distribution at greater depths provided by the minirhizotron approach.

## Conclusion

The methods demonstrated in this study represent a substantial increase in the scale of minirhizotron experimentation, and open up the possibility of using the method in studies that use quantitative genetic techniques to discover the phenotype-to-genotype associations as well as trait relationships that are current major knowledge gaps in root biology [2, 46, 55, 56].

## Methods

### Field site

Diverse populations of 369 inbred maize lines (2017) and 700 hybrid maize lines (2018 and 2019) were planted in replicated blocks on the experimental farms of the University of Illinois at Urbana‐Champaign with a precision planter. Each subplot containing a single genotype had four rows with length of 3.65 m, row spacing of 0.76 m, and plant spacing within a row of 0.18 m.

### Minirhizotron materials

Cellulose acetate butyrate (CAB) minirhizotron tubes of 183-cm (6 ft.) length, 5.715-cm diameter, wall thickness of 0.3175 cm, and internal diameter of 5.3975 cm were used (Exelon by Thermo, Georgetown, Delaware, USA). Tubes were sealed at the bottom with low-density polyethylene cap plugs (product # CCF-2 1/4-10-14, Caplugs, Buffalo, NY, USA; 5.7150 cm outer diameter) and glued with adhesive (Loctite Instant mix) and all-purpose non-toxic sealant (GE All Purpose Silicone 1) to avoid water infiltration from the soil into the tube. A small indexing hole was drilled near the top of the tube using a guide to maintain consistent position (Bartz, CA, USA) which helps in locking the camera to the tube during imaging. Tubes were stored in batches of 300 in collapsible containers (Uline product # H-1732), which had wooden separators for easy stacking and to avoid shifting during transport to the field by forklift.

Minirhizotron caps that prevented rain from entering the tube and excluded light from being transmitted below-ground by the tube were constructed from 6.35-cm diameter, schedule 40 PVC pipe. Each cap was 31 cm long, with one end closed by a socket cap (product # 447-25, Lasco Fittings Inc. Brownsville, TN, USA) and the other end cut at an angle to rest upon the soil surface. The caps were stored and transported in polypropylene bags with forklift straps that are designed for transport of building materials.

### Installation equipment

Four sets of hydraulic soil coring probes (Model 15GSRTS, Giddings Machine Co., Windsor, CO, USA) mounted to small tractors (Model 5065E with 512 front loader, John Deere, Moline, IL, USA; Fig. 1) were used to bore holes, insert minirhizotron tubes, and extract minirhizotron tubes. The tractors were fitted with 350 kg rear-wheel weights. Steel bars 2.5 cm thick, 10 cm wide and 183 cm long were custom fabricated and mounted to the front loader bucket to act as a physical brake against the forward force produced by the corer during installation (Fig. 1a). The spikes were tapered to aid insertion into the soil as a physical break and had several sets of mounting holes to allow length adjustment during transport and installation.

Each hydraulic corer was modified to include a telescoping tubular steel bar that maintained the mast at the desired angle of 30˚ from vertical during installation but still allowed the mast to be “laid down” when tubes are not being installed. A heavy-duty Kelly bar (KB-308) with a 5.715 cm soil tube adapter was used to mount the coring tube. A steel soil coring tube of long slotted or solid wall type (152 cm (5 ft.) length and 5.715 cm outer diameter) was fit with sharpened coring bits. This tube exactly matched the outer diameter of the minirhizotron tubes. The internal diameter of the bit was progressively reduced as tubes needed to be installed in wetter and heavier soils: from standard (4.7625 cm) in dry soils, to quick-relief (4.6037 cm), heavy quick relief (4.445 cm) and extra heavy quick relief (4.2735 cm) in wet, heavy clays. The use of slotted type soil corer tubes helped in avoiding compaction. Also, if coring tubes became clogged with soil, pry bars were inserted into the slit to clear the blockage.

### Installation technique

Two to four minirhizotron tubes were installed in each genotype subplot. The minirhizotrons were located centrally in each 4-row plot (Fig. 3). The locations were predetermined and marked with flags. The tractor was driven into the field straddling the two rows of crop where minirhizotron tubes were to be installed and stopped over the initial installation position. The front loader was lowered to insert the steel bars into the soil as a physical brake. The mast of the hydraulic corer was rotated on the turntable such that the coring tube would enter the soil directly within the row of crops. To avoid compression of soil, repeated short cores were taken with the use of multiple soil corer tubes to make each ~ 152-cm long hole (https://www.youtube.com/watch?v=kciMoWH-fFk). The soil from each excavated core was collected in a waste bucket and dumped at the edge of the field to avoid mixing of subsoil with topsoil in experimental plots. After the hole was cored out, a cap was placed over the minirhizotron tube and it was pushed into the hole by placing applying pressure from the hydraulic system to the top of the cap with the soil tube adapter. It was important to maintain steady, low pressure from the hydraulic system to ensure a smooth installation of tubes and to avoid bending or breakage. After the tube was installed, the mast of the hydraulic corer was swung over to allow installation of the next tube in the other middle row of crops, or driven forward to the next position for tube installation in the same row. After all minirhizotron tubes in a given plot were installed, the tractor was driven to the next plot and installation continued. The tractors always drove in a south to north direction so that all tubes had the same orientation. If all the tubes in a given range of plots were not completed within a day, the tractor was left in the field and installation continued from that point the next day. This minimized traffic through the plots to a single pass of the tractor in all cases.

### Minirhizotron angle and depth

A digital clinometer app on cell phones was used to measure the angle of installed minirhizotron tubes and the length of the tube protruding from the soil was measured with a measuring tape in order to subsequently calculate the vertical depth distribution of roots. Tubes were typically inserted to a depth of 152 cm, but on rare occasions (< 0.1% of all tubes) a tube could not be fully inserted. If tubes were installed to ≥ 75% of full length they were trimmed to the appropriate height above-ground and images collected from the portion of the tube that could be used. If tubes were installed < 75% of full length they were trimmed to the soil surface and dropped from the experiment.

### Image collection

Four minirhizotron video cameras (BTC100x, Bartz Technology Corp, Carpinteria, CA, USA) with incandescent illumination were used in parallel to capture digital color images of roots. The camera was attached to an indexing handle and controlled with a laptop computer-based image capture system (I-CAP, Bartz Technology Corp, Carpentaria, CA, USA). Prior to imaging roots, a reference grid (1 × 1 mm grid) was imaged after adjusting zoom, light intensity and the image perimeter area (2 cm wide) to standard settings that were maintained throughout data collection. The laptop and its 12 V battery power source were kept in a wheelbarrow, which made it easier for a single operator to move from plot to plot within the field over long periods of image collection.

Prior to imaging, each minirhizotron tube was checked for water and was cleaned with a long-handled swab for dust and water condensation inside the inner surface of the tube. Images were collected from the upward facing surface of the tube at increments of 1.3 cm down the length of the tube, and at a speed of 1200 ms per index handle location. The quality of images was monitored on the laptop. When images were identified as blurry or out of focus, that minirhizotron was re-imaged. The resulting images had a resolution of 28 µm per pixel and were each 640 × 480 pixels (18 × 13.5 mm) in size. Collectively, they record the distribution of root length across a range of depths of the soil profile (e.g. Fig. 2).

### Minirhizotron tube removal

At the end of the growing season, after all sampling was complete, the same tractor-mounted Giddings were used to pull the minirhizotron tubes out of the ground by attaching a Sentek access tube extraction tool (Fondriest Environmental – Fairborn, Ohio, USA) and chains bolted to the Kelly bar. This procedure was not particularly time sensitive and was typically completed at a rate of ~ 300 tubes per day with two operators for each of two tractors.

## Data Availability

Not applicable.
